# Workaholism in Korea: Prevalence and Socio-Demographic Differences

**DOI:** 10.3389/fpsyg.2020.569744

**Published:** 2020-12-23

**Authors:** Sudol Kang

**Affiliations:** College of Global Business, Korea University, Sejong-City, South Korea

**Keywords:** workaholism, work addiction, South Korea, demographic differences, logistic regression

## Abstract

This study has two objectives – to provide a Korean form of the workaholism analysis questionnaire, and to analyze workaholic tendencies in South Korea by using a nationally representative data. Using 4,242 samples (2,497 men and 1,745 women), exploratory and confirmatory factor analyses were conducted to develop a Korean form (K-WAQ). The four-factor structure of K-WAQ in this study seemed to adequately represent the underlying dimensions of work addiction in Korea. The study also analyzed the prevalence of workaholism among Koreans and its differences according to socio-demographic variables. Both mean difference analyses and logistic regressions were conducted. The overall result indicated that the prevalence of workaholism in Korea can be estimated to be 39.7% of the employees. The workaholic tendencies in Korea differ significantly according to gender, age, work hours, and voluntariness of choosing employment type. Practical as well as theoretical implications and future research directions are discussed.

## Introduction

The term workaholism, meaning addiction to work, was defined by Oates (1971, p.11) as the “compulsion or uncontrollable need to work incessantly.” It is now broadly accepted that workaholism is a form of behavioral addiction (Schaef, 1987; Robinson, 1998/2013; Andreassen et al., 2018; Griffiths et al., 2018) that has similar mechanisms and effects as substance addictions.

Despite some positive aspects of work itself (Machlowitz, 1980), work addiction is often characterized as a fatal disease (Fassel, 1990). Although several studies on workaholism have been conducted over the last five decades, there is still little consensus on the definition and the roots of workaholism (Andreassen et al., 2018; Griffiths et al., 2018; Atroszko et al., 2019).

The most commonly shared concept of workaholism is defined as a continual pattern of working excessively beyond expectations, and a compulsive obsession with work (Ng et al., 2007; Griffiths, 2011; Andreassen, 2014). In particular, Ng et al. (2007) underlined that the emotions, thoughts, and behaviors of workaholics are generally ruled by their work. Thus, they define workaholism as multidimensional, including affect, cognition, and behavior aspects in their theoretical model. The current study shares this view, as feelings, thoughts, and attitudes precede human actions, and the way we act is derived from what and how we feel, think, and believe.

The motives of compulsive dependency underlying workaholism are, *de facto*, multidimensional: lack of self-esteem, inferiority feeling, repeated experiences of traumatic incidents, fear of failure, desire for achievement, organizational pressure, and performance-oriented society (Killinger, 1991; Burke, 2000; Carroll and Robinson, 2000; Porter, 2001; Mudrack, 2006; Griffiths, 2011; Andreassen, 2014). Therefore, it is reasonable to regard workaholism as driven by internalized norms of self-worth as well as social approval (Ryan and Deci, 2000; Van Beek et al., 2012; Stoeber et al., 2013; Quinones and Griffiths, 2015).

Meanwhile, it is also significant to consider the *dynamic* character of workaholism. As work addiction is not a state but a *process*, it can take two directions, namely, going forward or being cured (Schaef and Fassel, 1988). Workaholics pursue increasingly higher performance to be gratified. When not working, they feel unstable, anxious, empty, and powerless (i.e., withdrawal symptoms). Therefore, without all-round efforts to recover from workaholism, the work-addicts tend to increase the quantity as well as the level of their work for higher gratification. In the illusion of control, they continue to ensure their work inventory ceaselessly. Although this aggravates their health as well as social relations, they try to tolerate the conflicts and problems ensuing from the addictive process.

It is noteworthy to examine why the definition of workaholism includes the aspect of the obsessive increase in work performance for gratification *despite* work–life imbalance and its negative consequences. Workaholic employees invest an abnormal amount of time and energy in work, but this behavior does not necessarily translate into enhanced organizational outcomes in the long term. Rather, workaholics display an impaired work performance resulting from the compulsiveness to make their work more complex than needed (Gorgievski and Bakker, 2010) and create more job demands for themselves because they were obsessed with unattainable standards and inclination to spend extraordinary time and efforts in unnecessary activities (Mazzetti et al., 2016).

Given this background, this study defines workaholism as a compulsive dependency on work despite harmful effects on health, social relations, and organizations. This definition includes all aspects of workaholism, such as affective, cognitive, attitudinal, and behavioral components (Ng et al., 2007) unlike other definitions that overemphasize just one or two aspects (cf. Spence and Robbins, 1992; Scott et al., 1997).

Although the labor productivity has increased enough for a substantial leisure society, workaholism nowadays passes for as a mass phenomenon in many modern societies (Schor, 1992; Heide, 2009). In fact, South Korea (since 1996) is the first among the Organisation for Economic Co-operation and Development (OECD) members with the longest working hours, and since 2008 it has been next to only Mexico. In 2014, the Koreans were reported to work 2,076 h, about 330 h more than the average (1,742 h) of OECD countries^1^. The Koreans worked about 350 h more than the Japanese. Compared to Norwegians or Germans, they worked in 2014 nearly 650∼700 h more.

One of the reasons such bizarre reality continued for a while is attributable to the work-oriented society as a whole since the rapid industrialization from 1960s. Moreover, although the newly revised Labor Standard Act (effective since July 2018) allows just 40-h week, and at most 12-h overtime per week if agreed by employees, the actual working time for permanent employees is often more than 60 h. Out of deep fear, people in Korea try to work as much as possible before losing their job (Kim and Lee, 2014). It is this fear emanating from the massive lay-off trauma in the wake of the Asian financial crisis (1997–2001) that compels them more severely to work addictively. However, studies investigating workaholism among Korean workers are extremely limited and rarely known in international academic circles. It is not only because the research on workaholism in Korea is mainly published in Korean (Kang, 2003; Han, 2011; Lee et al., 2015). Besides, there has been little evidence-based measurement adequate for assessment of workaholism in Korean culture.

It is against this backdrop that the current study was conducted. Thus this study aims, first, to provide a Korean measure (K-WAQ) of workaholism by modifying the original WAQ (cf. Kang, 2020), and secondly, to estimate the workaholism prevalence and its differences according to socio-demographic variables in South Korea.

## Psychometric Properties of K-WAQ and Its Validity

In the last decades many researchers have world-wide tried to develop measures of workaholism, as it was recognized as a harmful disease (Andreassen et al., 2018; Griffiths et al., 2018; Urbán et al., 2019). Two American measures, WorkBAT by Spence and Robbins (1992) and the WART by Robinson (1998/2013), have been widely used in assessing workaholism prevalence. However, both lacked validity and used limited samples, which in turn restricted their potential for generalization (McMillan et al., 2002). Besides, each measure had an unstable factor structure, validating only parts of the original components, two of three in the WorkBat (Andreassen et al., 2007), and three of five in the WART (Clark et al., 2010). Later two European measures such as Dutch Work Addiction Scale (Schaufeli et al., 2009) and Bergen Work Addiction Scale (Andreassen et al., 2012) were developed. The former is based both on the WorkBAT and the WART. And the latter is grounded in the components model of addiction (Brown, 1993; Griffiths, 2005). Although they are popular, they also have shortcomings, respectively. Schaufeli et al.’s (2009) DUWAS seems to have insufficient factor structure. Despite the advantage of its simple structure, the DUWAS is based on cognitive behavioral psychology rather than addiction theory. Although defining workaholics as a combination of excessive and compulsive workers (Schaufeli et al., 2009; Nonnis et al., 2017) distinguishes among relaxed, hard, compulsive, and workaholic employees, it does not necessarily provide an adequate equivalence to work-addicts. DUWAS fails to seize the underlying dynamic aspects of work addiction such as the illusion of control, the risk of relapse, and the endurance of conflicts by repressing fear. And the BWAS, despite its simplicity and convenience, runs the risk of having just one item as a single component of work addiction. Considering that one of the core feature of workaholics is denial, to measure each component of work addiction via a single item would be unreliable. Besides, as relevant researchers have suggested (MacCallum et al., 1999; Raubenheimer, 2004), we normally need at least three items to constitute one factor or subscale, in order to assess and diagnose work addiction as precisely as possible, unless there is strong theoretical backing for a less-than-three item factor design. Finally, the BWAS proposed a cut-off score for the judgment of workaholism (Andreassen et al., 2014): those responding positively to four or more of seven items are determined to be workaholics. Clinically (and also statistically) clear and useful as it is, this tool might mistakenly judge potential workaholics as healthy and sober. It is notable that workaholism as a behavioral addiction is a dynamic and progressive disease (Schaef, 1987; Fassel, 1990). Thus it is better to evaluate and measure workaholism as a continuum, not as a definite state or binary.

As indicated already by Kang (2020); Aziz et al. (2013) developed a comprehensive measure for workaholism, the workaholism analysis questionnaire (WAQ), in order to overcome these limitations. The WAQ is a 29-item self-reporting questionnaire that utilizes a 5-point Likert scale. In its conceptualization, it included items reflecting work–life imbalance, as this allegedly represents a common symptom of addictive disorders. An extensive literature review led Aziz et al. (2013) to derive five workaholism components: work–life conflict, work perfectionism, work addiction, unpleasantness, and withdrawal symptoms. They concluded that their WAQ provided stark content, concurrent, convergent, and discriminant validity as well as internal reliability.

Convinced as Aziz et al. (2013) were that the WAQ’s reliability and validity were confirmed, nevertheless, the WAQ seems to be problematic in terms of general applicability. Meanwhile the WAQ has been used in several research in America (Lanzo et al., 2016; Moyer et al., 2017; Aziz et al., 2018; Balkin et al., 2018). However, most measured the level of workaholism simply by the total scores of the scale without analyzing its factorial structure, i.e., irrespective of distinctive subscales. Besides, just one subscale, the work-life conflict, with seven items, was used in one study (Hamilton Skurak et al., 2018). This implies that the WAQ’s factor structure may be instable even in the US. In fact, the WAQ’s factorial structure was not persistent in some Korean research, each using different subset of the same data from the Korean Labor and Income Panel Study (KLIPS). For instance, Oum and Lee (2018) found a four-factor solution with 28 items from the WAQ, whereas Seo et al. (2018) obtained a six-factor structure with 25 items and Yoon (2018) reached another six-factor construct but with 28 items. In each case, both the factorial structure and the factor names were not consistently replicated. While Yoon (2018) used a subset (*N* = 6,254) of the full participants of the 17th KLIPS by excluding the data from students or unpaid family workers, Seo et al. (2018) analyzed 4,789 samples by deleting all the relevant data with missing values and Oum and Lee (2018) just 2,494 respondents of white collar workers under 60 of their age. In contrast, the current study utilized 4,242 participants by ruling out those non-typical data from unpaid family workers, the younger under 20, the elderly over 69 of their age, and some data with missing value. Table 5 shows also the representativeness of the sample in this study in comparison with national statistics on Korean workforce in the year 2014^2^. This suggests that the WAQ’s factor structure is not automatically applicable to the Korean population, and its lack of generalizability might be a considerable limitation. Thus it needs to come under systematic scrutiny, especially when applied to people with different cultures.

### Methods

In developing a Korean form of the WAQ it was necessary to translate the original 29-item WAQ into Korean language and to administer to Korean workers (Kang, 2020). The survey using the WAQ was conducted in the seventeenth wave of the KLIPS. Then exploratory factor analyses (EFA) were performed to extract appropriate parameters for a Korean form of WAQ (K-WAQ) using SPSS 21.0 with maximum likelihood (ML) estimation method. And with respect to the factor rotation, the method of oblique rotation was used. The reason for using the method of oblique instead of orthogonal rotation was that there might be certain correlations among sub-factors of the construct (Table 1). The oblique rotation can namely provide solutions with better simple structure (Fabrigar et al., 1999). When data are relatively normally distributed, ML is the best choice as “it allows for the computation of a wide range of indexes of the goodness of fit of the model [and] permits statistical significance testing of factor loadings and correlations among factors and the computation of confidence intervals.” (Fabrigar et al., 1999, p. 277). The relatively large size of the sample in this study (*N* = 4,242) enables the assumption that the data have a normal distribution. The discriminatory power of the items converging to each sub-scale is shown in Table 3 as a result of confirmatory factor analysis (CFA). For the validation of the K-WAQ, CFA was performed using AMOS 21.0 with ML estimation because of its statistical properties such as consistency, normality, maximal efficiency, and asymptotic unbiasedness (Li, 2016).

**TABLE 1:** Factor Extraction through EFA for K-WAQ (bold: factor loading >0.5).

Item (Factor Loading)	F1	F2	F3	F4	Factor Name
3. I feel anxious when I am not working.	**0.95**	−0.00	0.01	−0.03	1. Withdrawal Symptoms (WS) (EV = 6.2, α = 0.89)
4. I feel bored or restless when I am not working.	**0.85**	−0.03	0.02	0.06	
2. I feel guilty when I am not working.	**0.78**	−0.04	0.00	0.06	
5. I am unable to relax at home due to preoccupation at work.	**0.51**	−0.08	0.04	0.23	
7. I think about work constantly.	0.33	0.16	0.09	0.29	
1. I feel stressed out when dealing with work issues.	0.29	0.23	0.20	−0.15	
25. I often put issues in my personal life “on hold” because of work demands.	−0.01	**0.97**	−0.04	−0.10	2. Endurance of Work–
26. I often miss out on important personal activities because of work demands.	−0.00	**0.86**	0.05	−0.14	Life Conflict (EC) (EV = 1.7, α = 0.87)
24. My work often seems to interfere with my personal life.	0.02	**0.62**	0.15	0.04	
27. I find it difficult to schedule vacation time for myself.	0.01	0.50	0.00	0.03	
28. I have difficulty maintaining friendships.	−0.01	0.47	−0.07	0.26	
29. I have difficulty maintaining intimate relationships.	0.02	0.45	−0.06	0.32	
6. I constantly feel too tired after work to engage in non-work activities.	0.28	0.41	0.03	0.02	
19. I frequently check my work many times before I finish it.	−0.01	−0.00	**0.82**	−0.15	3. Illusion of Control (IC) (EV = 1.5, α = 0.79)
18. I often obsess about goals or achievements at work.	0.03	0.02	**0.68**	0.05	
22. It takes me a long time to finish my work because it must be perfect.	0.02	0.07	**0.65**	0.06	
20. I ask others to check my work often.	−0.06	0.04	**0.54**	0.25	
9. I have a need for control over my work.	0.10	0.01	0.44	−0.11	
10. I have a need for control over others.	0.03	−0.06	0.41	0.08	
14. I find myself unable to enjoy other activities because of thoughts of work.	−0.01	0.03	0.16	**0.79**	4. Compulsive Dependency on Work (CD) (EV = 1.2, α = 0.83)
12. I frequently have work-related insomnia.	0.09	0.17	0.11	**0.72**	
13. I feel very addicted to my work.	0.12	0.15	0.05	**0.69**	
15. I consider myself to be a very aggressive person.	0.09	−0.07	−0.08	**0.61**	
11. I enjoy spending evenings and weekends working.	0.16	0.16	0.12	0.48	
23. I experience conflict with my significant other or with close friends.	−0.01	0.23	0.08	0.47	
8. I prefer to work excessive hours, preferably 60 hours or more per week.	0.15	−0.06	−0.07	0.46	
16. I get irritated often with others.	0.01	0.07	0.28	0.42	
17. People would describe me as being impatient and always in a hurry.	0.06	0.05	0.33	0.40	
21. I frequently feel anxious or nervous about my work.	0.14	0.13	0.29	0.36	

*KMO = 0.919, χ^2^ = 1,423.5, d.f. = 89, *p* < 0.001. Factor Extraction Method = Maximum Likelihood, Rotation Method = Oblique (Direct Oblimin) with Kaiser Normalization, Total Variance Explained = 61.0%, Cronbach’s α = 0.895 (N of items = 15, *p* < 0.01). EV, Eigen value.*

#### Sample

This study used a sample of 4,242 respondents (with age between 20 and 69) out of the 17th KLIPS (Kang, 2020), as it performed an Additional Survey (AS) on time use and quality of life, including the 29-item WAQ by Aziz et al. (2013). All the data from KLIPS, except for respondents’ personal identification, are provided free to the public^3^ in the intention to promote policy developments as well as non-commercial research. The original 7,199 respondents of the 17th KLIPS could not be used in this study, as many had non-typical characteristics. Following samples were systematically excluded to enhance statistical effectiveness: first, the samples from students or unpaid family workers, second, those under 20 or over 70 years of age, and third, samples with missing values in relevant variables. This data-cleaning process reduced the number of effective samples in the current study to 4,242 samples.

About 36% of the respondents were factory workers, 27% professionals, 20% clerical staff, and 17% service employees. There were 1,745 females (41.1%) and 2,497 males (58.9%), and their mean age was 39. Table 5 shows the demographic characteristics and workaholism prevalence of the sample respondents. Besides, the Table 5 also indicates that the samples of this study present non-significant differences in its proportionality in comparison with the national workforce data of the Korean Statistical Bureau^4^.

#### Measures

##### Socio-Economic Demographics

The questionnaire included variables such as gender, age, annual income, employment type, marital status, work hours, and occupational sector.

##### Workaholism

The K-WAQ is a self-reporting questionnaire utilizing a five-point response format. Sample items include, “I enjoy spending evenings and weekends working” and “I often obsess about goals or achievements at work.” The K-WAQ used translation-back-translation to evaluate workaholism in Korea. This translation-back-translation method obtained items closer to the original scale in semantic relevance as well as content similarity. Slightly differently from the self-reporting method of the original WAQ, more than 95% respondents in this study selected, through face-to-face interviews, from the Likert scale ranging from 1 (strongly disagree) to 5 (strongly agree). Larger scores indicated higher levels of workaholic tendencies. Cronbach’s α was 0.93 in the present study.

##### Affective Commitment

In the current study, four questions were adopted to evaluate the respondents’ level of affective commitment (AC) to organization, which was originally developed by Porter et al. (1974). The AC already proved to be discriminantly valid (Aziz et al., 2013). Thus, AC was included into correlation analysis with four sub-scales of the K-WAQ in order to test discriminant validity of the K-WAQ. Response options were provided on a five-point scale, from 1 (strongly disagree) to 5 (strongly agree), with higher scores indicating higher levels of AC. The four items were “This is a good workplace to work at,” “I am glad to have joined this company,” “I would recommend joining this workplace to a friend searching for a job,” and “I take pride in being a part of this company.” Cronbach’s α of the uni-dimensional construct (AC) was 0.89 in this study.

### Results

#### Psychometric Properties of the K-WAQ

The K-WAQ was developed by conducting EFAs on the WAQ’s 29-items as well as analysis of item discrimination index (Kang, 2020). As a result, this study could finally extract 15 items adequate and appropriate for evaluating workaholism in Korea. At first, this study could eliminate four items (8, 9, 10, and 11) from the original WAQ, as these showed relatively low item discrimination indices, as recommended by D’Sa and Visbal–Dionaldo (2017). Further, ten items could additionally be excluded through EFA, based on comparatively low pattern coefficients (RobersonIII, Elliott et al., 2014). This pattern coefficients, i.e., factor pattern matrix loadings are the linear combinations of the factors that make up the original standardized variables. According to RobersonIII, Elliott et al. (2014), items with pattern coefficients larger than.50 can determine the saliency of significant items with each factor. It is notable that just one item (“I find it difficult to schedule vacation time for myself.”) indicated the factor loading of.50 converging to the factor of Endurance of Work–Family Conflict (EC). This could either be included into or excluded from the new construct (K-WAQ). However, it was not included in this study, just as other irrelevant items, in consideration of simplicity principle in social sciences (Feldman, 2016).

This study additionally conducted EFAs with three different subsets from the full sample to examine the stability of factorial structure. The first subset consisted of the first half of it and the second one the latter half, and the third one comprising randomly selected 50% of the full dataset by way of the case selection tool of SPSS. The results indicate, as shown in Tables 1 and 2, that the four-factor structure is to replicate in a stable way. All these procedures allowed the present study to preliminarily obtain a four-factor solution with 15 items. The Cronbach’s α, which shows the internal consistency of the scale, was 0.895. The split-half reliability coefficient of the new scale was 0.72 (Table 1).

**TABLE 2:** Result of EFA with three subsets of the full sample.

Items and Factor	EFA1 (first half)	EFA2 (second half)	EFA3 (random 50%)
i3	0.918	0.918	0.929
i4	0.830	0.830	0.836
i2 (F1 = WS)	0.760	0.760	0.760
i5	0.551	0.551	0.552
i25	0.921	0.921	0.919
i26 (F2 = EC)	0.912	0.912	0.905
i24	0.642	0.641	0.651
i19	0.805	0.805	0.811
i18	0.719	0.719	0.705
i22 (F3 = IC)	0.618	0.618	0.643
i20	0.522	0.522	0.538
i14	0.620	0.619	0.604
i12	0.563	0.563	0.568
i13 (F4 = CD)	0.546	0.545	0.562
i15	0.537	0.537	0.560
Nr. of items = 15	χ^2^ = 6,476.8, d.f. = 296, *p* < 0.001	χ^2^ = 6,473.8, d.f. = 296, *p* < 0.001	χ^2^ = 3,451.3, d.f. = 296, *p* < 0.001

**TABLE 3:** Convergent reliability and internal consistency of the new construct.

Factor → Item	B	s.e.	C.R.	β	AVE	Construct reliability	Cronbach’s Alpha
WS → 5	1.00			0.72	0.77	0.93	0.89
WS → 2	1.13	0.02	48.58	0.78			
WS → 4	1.29	0.02	53.93	0.86			
WS → 3	1.41	0.03	55.87	0.90			
IC → 20	1.00			0.63	0.58	0.85	0.79
IC → 22	1.31	0.04	36.03	0.73			
IC → 18	1.46	0.04	36.56	0.74			
IC → 19	1.52	0.04	35.27	0.70			
CD → 15	1.00			0.62	0.72	0.91	0.83
CD → 13	1.46	0.04	39.68	0.80			
CD → 12	1.36	0.04	37.82	0.74			
CD → 14	1.31	0.03	39.71	0.80			
EC → 24	1.00			0.72	0.75	0.90	0.87
EC → 26	1.21	0.02	52.98	0.86			
EC → 25	1.35	0.03	54.33	0.92			

*B, unstandardized regression weights; s.e., standard error; C.R., critical ratio > 1.96; β > 0.5; AVE (average variance extracted) > 0.5; construct reliability > 0.7.*

In sum, the final format of the measure, the K-WAQ, was derived depending on the factor loadings and the experts’ views (Kang, 2020). The content validity of the K-WAQ was reconfirmed through a strict examination by the five experts. Similar to the views of many researchers (Scott et al., 1997; Flowers and Robinson, 2002; Ng et al., 2007), factors such as compulsive dependence on work (CD), the illusion of control (IC), Endurance of work–family conflict (EC), along with withdrawal symptoms (WS), were extracted as essential factors to explain workaholism in Korea. While CD and WS represent the quintessential aspects of workaholism, as workaholism dynamically progresses (Schaef, 1987), both IC and EC show up more intensively, and much stronger (Fassel, 1990; Robinson, 1998/2013; McMillan et al., 2001). Thus the four-factor structure of workaholism in the present study seemed to adequately represent the underlying dimensions of work addiction.

#### Convergent and Discriminant Validity of the K-WAQ

Further, this research conducted CFA using a structural equation model (SEM) on the above 15 K-WAQ items to determine the validity and reliability of the new construct. As implied, the four-factor structure of K-WAQ seemed quite different from the five-factor model of Aziz et al. (2013) in the United States and proved to be better from a statistical perspective (cf. Table 5).

The CFA produced the following results. Although CMIN/DF (= 15.946, *p* < 0.001) was larger than 3, all the indices for model-fit (cf. Table 5) indicated that this measurement model was statistically appropriate and acceptable. Table 3 illustrates that all the standardized regression weights (β) were more than 0.5 (β > 0.5), all the average variance extracted (AVE) were larger than 0.5 (AVE > 0.5), and all the construct reliabilities were greater than 0.7. The AVE provides an estimate of how much of the item variance arises from the concerning construct. The construct reliability is equivalent to Cronbach’s alpha coefficient for the overall reliability of the items in the model (Pratarelli and Browne, 2002). Consequently, the convergent reliability, as well as internal consistency, of the workaholism construct was statistically confirmed.

Simultaneously, the present study examined the discriminant reliability of the construct, by comparing AVE and correlation coefficients (ρ) of the factors. All the AVE were greater than the square of ρ, validating the discriminant reliability of the construct (Table 4).

**TABLE 4:** Correlations among factors and discriminant validity test.

Factor (AVE)	CD	IC	EC	AC	Maximum ρ^2^
CD (0.715)				−0.103	
IC (0.583)	0.537*			−0.024	0.288
EC (0.751)	0.493*	0.465*		−0.132	0.243
WS (0.767)	0.646*	0.500*	0.387*	−0.081	0.417

* *p* < 0.05; (AVE) > ρ^2^. CD, compulsive dependency; IC, illusion of control; EC, endurance of conflicts; WS, withdrawal symptoms; AC, affective commitment; AVE, average variance extracted.

**TABLE 5:** Model comparison among different factor structure models.

Model	TVE*	χ^2^(CMIN)	df	RMR	GFI	AGFI	NFI	TLI	CFI	RMSEA
1	52.8%	14,238	367	0.056	0.767	0.724	0.766	0.746	0.771	0.094
2	61.0%	1,339	84	0.031	0.960	0.942	0.959	0.952	0.962	0.059
3	41.4%	133.8	90	0.079	0.679	0.572	0.633	0.573	0.634	0.177
4	61.0%	992.1	84	0.033	0.957	0.939	0.957	0.950	0.960	0.060

*Model 1: 5-factor (WAQ, *N* = 4,242), Model 2: 4-factor (K-WAQ, *N* = 4,242), Model 3: Single-order (K-WAQ, *N* = 4,242), Model 4: 4-factor (*N* = 2,981). TVE, total variance explained.*

#### Discriminant Validity Test: Correlations Between K-WAQ Subscales and AC

As Table 4 shows, the present study examined the correlation between the four dimensions of the K-WAQ, i.e., CD, IC, EC, and WS, and one of the relevant but differential variables from workaholism construct, the AC to organizations (Porter, 1996; Scott et al., 1997; Buelens and Poelmans, 2004; Aziz et al., 2013). AC refers to the level of emotional attachment and identification with the organization (Porter et al., 1974), which is, according to Aziz et al. (2013), quite different from the workaholism construct as a behavioral addiction. Porter (1996) also stressed that workaholics are often isolated, incapable of team playing, and rigid in thinking and acting. Precedent research reassured these two constructs are theoretically distinctive (Scott et al., 1997; Buelens and Poelmans, 2004). Table 4 shows extremely low correlation coefficients (0.02 < |ρ| < 0.13) between four factors of K-WAQ and AC. Discriminant validity is established when measures of different constructs are proven to be uncorrelated with each other (Campbell and Fiske, 1959; Zaiţ and Bertea, 2011). Therefore, the results in Table 4 provide evidence that the K-WAQ practically has discriminant validity.

Furthermore, this study tried to compare between four-factor structure of the current research and other models in order to test the construct validity. Table 5 demonstrates the differences in model-fit indices among various models. Compared to the Model 1 of Aziz et al. (2013), the four-factor model of the current study (Model 2) fits much better in measuring workaholism in Korea (cf. Table 5). Moreover, this Model 2 with four-factor structure was replicated in Model 4 with different subsamples (*N* = 2,981) that was randomly selected. Lastly, it became clear that Models 1, 2, and 4 with second-order structure showed definitely far better model fit than single-order structure (Model 3). Especially the comparison between Model 2 and Model 3 indicates that two-dimensional structure measures workaholism in Korea much better than uni-dimensional one. Therefore, this study come to the conclusion that the K-WAQ, comprising 15 items and four sub-scales, can function as a suitable measure for workaholism in Korea.

## Prevalence of Workaholism in Korea

In the second phase of this study, based on the K-WAQ, the prevalence and differences of workaholism among employees in South Korea were investigated.

### Methods

#### Socio-Demographics

For this study, some socio-demographic data, except for gender and voluntariness in choosing employment type, were stratified. First, regarding job security, the full-time workers with job security and direct labor contract with employers were defined as permanent. Second, regarding marital status, those living with a partner and without a partner, for any reason, were differentiated. Third, ages were stratified into five groups such as 30 > G1 ≥ 20 and 70 > G5 ≥ 60. Fourth, yearly household income levels were clustered into five groups in the same manner. For instance, $ 20,000 > G1, $ 60,000 > G3 ≥ $ 40,000, and G5 ≥ $ 80,000. Fifth, actual weekly work hours were also classified into five groups, such as 30 hours > G1, 50 hours > G3 ≥ 40 hours, and G5 ≥ 60 hours. Sixth, educational attainment levels were also grouped into five, middle school ≥ G1, university > G3 ≥ college, and G5 ≥ graduate school.

#### Workaholism

To evaluate the prevalence of workaholism in Korea, this study used K-WAQ – validated in the first step of this study – which comprises 15 items with four factors: CD, IC, EC, and WS. Workaholism can be measured either as a continuum or as a binary. As a continuum the average score of the 15-items was used to examine the differences of workaholism level. Besides, a score of 4 (agree) or 5 (strongly agree) on at least eight of the 15 items was recommended as a cut-off for workaholism being present in workaholics [cf. Lemmens et al., 2009; American Psychiatric Association (APA), 2013; Andreassen et al., 2014].

#### Statistics

Descriptive statistics of the distribution of nominal or classified variables were computed to better understand the sample. The average score of K-WAQ was compared among various groups of the nominal or stratified variables (gender, age, income, marital status, education, job security, work hours, and voluntariness in choosing employment type) by *t*-tests or one-way ANOVA. In addition, a General Linear Model (GLM) was used to detect the interaction effect of gender and marital status with workaholism. The prevalence of workaholism (with 95% confidence interval) in Korea was calculated. A sensitivity analysis concerning different cut-offs (scoring 4 or 5 on 1 to 15 items) was also conducted.

Finally, logistic regression analyses were conducted to compare the effects of relevant factors on workaholism (1 = workaholic and 0 = non-workaholic) in Korea. The independent variables were the eight socio-demographic variables. The dependent variable was set using the above-mentioned cut-off point. The odds ratio (OR), named as Exp(B) in Table 11, is to be interpreted as significant if the 95% confidence interval does not include 1.00.

### Results

Table 6 provides descriptive statistics of the sample and the workaholism magnitude in South Korea according to the eight socio-demographic variables. The scores of workaholism in Table 6 indicate, for each group, the average level of K-WAQ, the 15-item measure of workaholism in Korea. The average score of K-WAQ for the whole sample (*N* = 4,242) was 2.31 and the standard deviation was 0.51.

**TABLE 6:** Socio-demographic characteristics and workaholism differences in Korea.

Category	Group	Nr.	%	2014 – National Workforce (%)	K-WAQ	*t*-tests, ANOVA
Gender	Male	2,497	58.9	57.7	**2.33**	*t* = 3.91
	Female	1,745	41.1	42.3	2.27	(*p* < 0.05)
Age	I. 20 ≤ Age < 30	476	11.2	11.8	2.28	
	II. 30 ≤ Age < 40	1,302	30.7	28.1	**2.34**	II, III > IV, V
	III. 40 ≤ Age < 50	1,245	29.3	26.9	**2.33**	
	IV. 50 ≤ Age < 60	869	20.5	22.9	2.26	(*F* = 5.64,
	V. 60 ≤ Age < 70	350	8.3	10.3	2.25	*p* < 0.05)
Annual Income (Thous. US Dollars)	I. AI ≤ 20,000	928	21.9	32.0	2.33	
	II. 20,000 < AI ≤ 40,000	1,887	44.5	38.2	2.29	
	III. 40,000 < AI ≤ 60,000	965	22.7	22.3	2.33	n.s.
	IV. 60,000 < AI ≤ 80,000	305	7.2	5.4	2.32	
	V. 80,000 < AI	157	3.7	2.1	2.28	
Job Security	Permanent	2,930	69.1	64.6	**2.32**	*t* = 3.24
	Non-permanent	1,312	30.9	35.4	2.27	(*p* < 0.05)
Marital Status	Not-living with partner	1,194	28.1	34.7	2.29	
	Living with partner	3,048	71.9	65.3	2.31	n.s.
Actual Work Hours per week	I. WH < 30	242	5.7	4.9	2.14	
	II. 30 ≤ WH < 40	2,577	60.7	50.3	2.31	IV, V > I, III,
	III. 40 ≤ WH < 50	846	19.9	21.2	2.28	III, II > I
	IV. 50 ≤ WH < 60	378	8.9	17.4	**2.39**	(*F* = 11.84,
	V. 60 ≤ WH	199	4.7	6.2	**2.41**	*p* < 0.05)
Education Level	I. EL ≤ middle school	483	11.4	17.7	2.26	
	II. EL ≤ high school	1,396	32.9	39.2	2.27	IV > I, II
	III. EL ≤ technical college	833	19.6	18.1	2.30	
	IV. EL ≤ university	1,257	29.6	20.4	**2.36**	(*F* = 6.91,
	V. EL ≥ graduate school	273	6.4	4.6	2.34	*p* < 0.05)
Voluntariness in Employ	Voluntary	3,369	79.4	n.a.	2.30	*t* = −2.25
	Involuntary	873	20.6	n.a.	**2.34**	(*p* < 0.05)
Total		4,242			2.31 (s.d. = 0.51)	Min. = 1.00, Max. = 4.73

**Source:* National Workforce Survey in 2014 at http://kostat.go.kr, retrieved on September 2, 2020. Bolded values mean that respective sub-group(s) show(s) more workaholic than other sub-group(s) with statistical significance.*

As with the K-WAQ the cut-off was set, when answering eight questions out of 15 with a 4 or 5 indicated that the person is likely to be a work-addict (Andreassen et al., 2014; Aldahadha, 2019). This cut-off was adopted from previous research in behavioral addiction and the fifth DSM [World Health Organization (WHO), 1992; Lemmens et al., 2009; American Psychiatric Association (APA), 2013]. Thus, the cut-off was 36 in sum of the K-WAQ, which was a mean of 2.40. Therefore, the mean value of 2.40 and above on the total scale indicated workaholism in Korea. Results showed that the prevalence of workaholism was 39.7% (95% CI = 38.2∼41.3%). To the best knowledge of the current study, this prevalence rate is the highest, at least, among the reported research on workaholism.

According to the results of t-tests and ANOVA, the Korean workers’ workaholic tendencies did not differ significantly according to marital status *per se* or income levels. On the contrary, statistically significant differences were detected according to gender, age, job security, voluntariness in employment, education levels, and work hours. More specifically, men (mean = 2.33, sd = 0.50; *t* = 3.91, *p* < 0.05) were more workaholic than women (mean = 2.27, sd = 0.53), and permanent workers (mean = 2.32, sd = 0.51; *t* = 3.24, *p* < 0.05) more than non-permanents (mean = 2.27, sd = .51). Interestingly, those who voluntarily (mean = 2.30, sd = 0.51; *t* = −2.25, *p* < 0.05) took their job type (either permanent or non-permanent) showed less workaholism than the involuntary job takers (mean = 2.34, sd = 0.51).

Moreover, ANOVA and *post hoc* multiple-comparison analyses by Scheffe Test revealed that among some sub-groups in age, education, and actual work hours, statistically significant differences existed. For instance, the age groups II (the 30s; mean = 2.34, sd = 0.51, *p* < 0.05) and III (the 40s; mean = 2.33, sd = 0.52, *p* < 0.05 showed statistically more workaholism than the groups IV (the 50s; mean = 2.26, sd = 0.50) and V (the 60s; mean = 2.25, sd = 0.54) [*F* = 5.64, *p* < 0.05]. The education level IV (university) group (mean = 2.36, sd = 0.51, *p* < 0.05) showed more workaholism than group I (middle school; mean = 2.26, sd = 0.54) and II (high school; mean = 2.27, sd = 0.51) [*F* = 6.91, *p* < 0.05]. Finally, the longest working group V (m = 2.41, sd = 0.53, *p* < 0.05), working 60 or more hours per week, or group IV (m = 2.39, sd = 0.53, *p* < 0.05), working 50 h and more but less than 60 h per week, displayed more workaholism than groups I (m = 2.14, sd = 0.49), working less than 30 hours week and III (m = 2.28, sd = 0.54), working 30 h and more but less than 40 h per week [*F* = 11.84, *p* < 0.05].

Interestingly enough, an analysis using GLM revealed that the interaction between gender and marital status has a significant impact on the level of workaholism (Table 7), while the same effect between voluntariness in employment and work hours does not exist. As shown in Table 8, men living with a married partner are highest on the K-WAQ followed subsequently by women living alone, women living with a married partner, and men living alone (*F* = 5.14, *p* < 0.05).

**TABLE 7:** Tests of between-subjects effects by GLM (interaction effect).

Source	Type III sum of squares	d.f.	Mean square	*F*	Sig.
Gender	0.046	1	0.046	0.348	0.555
Marital Status	0.163	1	0.163	1.235	0.266
G × M	0.680	1	0.680	5.141	0.023

**R*^2^ = 0.288, adjusted *R*^2^ = 0.278.*

**TABLE 8:** Descriptive data on the interaction effect of gender and marital status.

Gender	Marital status	Mean	S.E.	95% CI	
				
				LL	UL
Female	Without partner	0.163	0.015	0.134	0.193
	With partner	0.149	0.011	0.128	0.170
Male	Without partner	0.127	0.015	0.099	0.156
	With partner	0.170	0.008	0.153	0.186

Besides, a sensitivity analysis was conducted for different cut-offs (scoring 4 or 5 on items 1 to 15). This analysis illustrated that the workaholism prevalence rate in Korea can range from 100% (scoring 4 or 5 on one item only) to 0.0% (scoring 4 or 5 on all 15 items). The results are presented in Table 9.

**TABLE 9:** Sensitivity for different cut-offs based on the K-WAQ.

Nr. of items with a score of 4 (often) or 5 (always)	Number of respondents	Estimated prevalence	95% Confidence interval
1 item	4,242	100%	100∼100%
2 items	4,242	100%	100∼100%
3 items	4,242	100%	100∼100%
4 items	4,182	98.6%	98.2∼98.9%
5 items	3,999	94.3%	93.6∼95.0%
6 items	3,783	89.2%	88.3∼90.1%
7 items	2,645	62.4%	60.9∼63.7%
8 items	**1,686**	**39.7%**	**38.2∼41.3%**
9 items	895	21.1%	19.8∼22.4%
10 items	475	11.2%	10.2∼12.1%
11 items	163	3.8%	3.2∼4.5%
12 items	57	1.3%	1.0∼1.7%
13 items	11	0.3%	0.1∼0.4%
14 items	2	0.0%	0.0∼0.1%
15 items	1	0.0%	0.0∼0.1%

*Bolded value means the estimated prevalence of workaholism in Korea.*

Further, Table 10 provides the proportion of those manifesting each of the 15 workaholism criteria of K-WAQ (i.e., scoring 4 or 5). This ranged from 2.9% (WAQ Item 15) to 27.2% (WAQ Item 19).

**TABLE 10:** Percentage of respondents endorsing (scoring 4 or 5) each item of K-WAQ.

Item	Wording	Addiction component	Percentage (95% CI) scoring 4 or 5
2	I feel guilty when I am not working.	Withdrawal Symptoms	8.1%(7.3∼9.0%)
3	I feel anxious when I am not working.		11.5%(10.6∼12.5%)
4	I feel bored or restless when I am not working.		9.4%(8.6∼10.3%)
5	I am unable to relax at home due to preoccupation at work.		6.2%(5.5∼6.9%)
12	I frequently have work-related insomnia.	Compulsive Dependency on Work	5.4%(4.7∼6.0%)
13	I feel very addicted to my work.		5.6%(4.9∼6.3%)
14	I cannot enjoy other activities because of thoughts on work.		3.3%(2.7∼3.8%)
15	I consider myself to be a very aggressive person.		2.9%(2.4∼3.4%)
18	I often obsess about goals or achievements at work.	Illusion of Control	14.5%(13.4∼15.5%)
19	I frequently check my work before I finish it.		27.2%(25.8∼28.6%)
20	I ask others to check my work often.		5.5%(4.8∼6.2%)
22	It takes me a long time to finish work as it must be perfect.		10.5%(9.5∼11.4%)
24	My work often seems to interfere with my personal life.	Endurance of Work–Life Conflict	13.9%(12.9∼14.9%)
25	I often put my personal life “on hold” because of work.		19.5%(18.3∼20.7%)
26	I often miss important personal activities because of work.		17.5%(16.4∼18.6%)

**TABLE 11:** Logistic regression for workaholism in Korea.

Variables	Group	Crude OR	Adjusted OR
Gender	Female	0.74** (0.66–0.84)	0.83** (0.72–0.94)
	Male	1.00	1.00
Age	I. 20 ≤ Age < 30	1.10 (0.83–1.47)	1.12 (0.79–1.59)
	II. 30 ≤ Age < 40	1.40** (1.10–1.80)	1.32 (0.98–1.77)
	III. 40 ≤ Age < 50	1.45** (1.13–1.86)	1.46** (1.10–1.93)
	IV. 50 ≤ Age < 60	1.18 (0.91–1.53)	1.23 (0.94–1.62)
	V. 60 ≤ Age	1.00	1.00
Annual Income (in thousand US Dollars)	I. AI ≤ 20,000	1.13 (0.80–1.59)	1.34 (0.92–1.93)
	II. 20,000 < AI ≤ 40,000	0.95 (0.68–1.32)	0.98 (0.70–1.56)
	III. 40,000 < AI ≤ 60,000	1.03 (0.73–1.46)	1.08 (0.76–1.53)
	IV. 60,000 < AI ≤ 80,000	1.04 (0.70–1.54)	1.05 (0.70–1.56)
	V. 80,000 < AI	1.00	1.00
Job Security	Non-Permanent	0.81** (0.71–0.93)	0.90 (0.76–1.05)
	Permanent	1.00	1.00
Marital Status	Not-Living with a partner	0.89 (0.78–1.02)	0.86 (0.72–1.01)
	Living with a partner	1.00	1.00
Actual Working Hours per week	I. WH < 30	0.34** (0.23–0.51)	0.35** (0.23–0.53)
	II. 30 ≤ WH < 40	0.63** (0.47–0.83)	0.56** (0.41–0.75)
	III. 40 ≤ WH < 50	0.56** (0.41–0.77)	0.53** (0.39–0.73)
	IV. 50 ≤ WH < 60	0.89 (0.63–1.26)	0.89 (0.63–1.26)
	V. 60 ≤ WH	1.00	1.00
Education Level	I. EL ≤ middle school	0.76 (0.56–1.03)	0.79 (0.55–1.12)
	II. EL ≤ high school	0.79 (0.61–1.03)	0.71* (0.53–0.94)
	III. EL ≤ college	0.91 (0.69–1.20)	0.90 (0.67–1.20)
	IV. EL ≤ university	1.09 (0.84–1.43)	1.08 (0.82–1.42)
	V. EL ≥ graduate school	1.00	1.00
Voluntariness in Employment	Involuntary	1.25** (1.08–1.46)	1.36** (1.16–1.59)
	Voluntary	1.00	1.00

*p < 0.05, **p < 0.01, Adjusted Regression Model χ^2^ = 122.71, df = 20, *p* < 0.001.Hosmer-Lemeshow χ^2^ = 5.81, df = 8, *p* = 0.67.

Table 11 shows the outputs from the binary logistic regressions in terms of OR and 95% confidence intervals for both the adjusted and crude analyses. For all the eight dummy coded variables, the last group comprises the reference (for which the OR equals 1.00). In both the crude and adjusted analyses, workaholism was significantly and positively associated with male workers, compared to the female group. The same is applicable to work hours, age, and voluntariness in employment. Those groups working less than 50 h per week were compared to the reference group (working 60 h and more) were significantly and negatively associated with workaholism both in the crude and adjusted analysis. Similarly, those who were involuntary job takers had a significant and positive possibility of workaholism in both analyses, compared to the those who chose their job voluntarily. Also those aged 40∼49 years were positively related to workaholism in both regressions, compared to those in their 60s. In contrast, in the crude analysis, those aged 30∼39 years were significantly and positively associated with workaholism, but this did not remain significant in the adjusted analysis. The same applies to job security. Those with permanent jobs had a significant positive relationship with workaholism in the crude analysis, although no significant association was found in the adjusted analysis. On the contrary, there was no significant relationship between education level and workaholism in the crude analysis, but in the adjusted analysis, those with high-school education showed a significant negative association with workaholism compared to those with graduate school attainment. Interestingly, in relation to household income, workaholism was unrelated to any of the income levels both in the crude and adjusted analysis. The same is valid for the marital status *per se*.

The full model, including all predictors for the logistic regression, proved statistically significant (χ^2^ = 122.71, df = 20, *p* < 0.001). Furthermore, the overall logistic model was statistically optimal (Hosmer-Lemeshow χ^2^ = 5.81, df = 8, *p* = 0.67) and explained between 2.9% (Cox and Snell *R*^2^) and 3.9% (Nagelkerke *R*^2^) of the variance in workaholism status (K-WAQ ≥ 2.4) and correctly classified 61.0% of all the cases. Interestingly, the effect of job security on workaholism prevalence disappeared in the full model.

### Discussion

Based on the K-WAQ, this study aimed to examine the prevalence of workaholism in a nationally representative sample of Korean employees. The prevalence of workaholism assessed as a behavioral addiction in line with Andreassen et al. (2014) and Lemmens et al. (2009) was estimated to 39.7%.

This workaholism prevalence in Korea seems somehow extreme. However, considering the 8.3% of workaholism in Norway (Andreassen et al., 2014), it is not an overestimation to predict that the workaholism prevalence in Korea amounts to 39.7%, where workers work annually, on average, 650 hours more than in Norway. Some Korean researchers have estimated the prevalence of workaholism in Korea ranging from 7.0% (Yoon, 2018) up to 15.1% (Seo et al., 2018). They used the same data source from the KLIPS, however, the samples and workaholism criteria used in the respective research were different. For example, Seo et al. (2018) analyzed their samples (*N* = 4,789), in exclusion of all data with missing values, and came to the conclusion that about 15% of the participants belong to performance-oriented workaholism group, whereas Yoon (2018) estimated the workaholism prevalence by calculating the average score of the 27 items and by examining if the score is larger than three. The analysis object of Oum and Lee (2018) was just white collar workers and they mainly examined, without providing any information on workaholism prevalence, the relationships among job insecurity, satisfaction with family relationship and workaholism. According to the results of the current study, nevertheless, it might have been underestimated. Although actual work hours are not a direct indicator of workaholism, its correlation coefficient to workaholism in most research has proven positive and significant (Aziz and Zickar, 2006; Schaufeli et al., 2008). To date, many researchers have reported on workaholism prevalence in each country, though with different methodologies and samples: 27% in Canada (Kemeny, 2002), 25% in the United States (Robinson, 1998/2013), 21% in Japan (Kanai et al., 1996), 13.8% in Iran (Ariapooran, 2019), 10% in the United States (Sussman et al., 2011), 8.3% in Norway (Andreassen et al., 2014), 7.6% in Italy (Villella et al., 2011), and 6.0% in Poland (Kunecka and Hundert, 2019). With this international magnitude of workaholism in mind, even the estimate of 39.7% in South Korea cannot be simply dismissed as exorbitant. To take this reality with earnest, the whole society of Korea should be aware how serious the workaholism level of the nation is.

We need some explanations about this high workaholism prevalence in Korea. First, the traumatic processes of industrialization since the Japanese colonialism have resulted in deep collective trauma in the whole Korean society (Heide, 2001). The collective trauma led the whole society to identification with work-oriented system as a survival strategy (Heide, 2009). The spectacular economic growth in Korea from 1960s up to 1980s, and up to 1997, before the Asian financial crisis, was in fact a product of this workaholic society (Kang, 2000). Second, people in Korea are growing up from childhood by education system with the values of diligence and loyalty. Young students are learning in kindergartens and schools to work hard according to the guidelines of teachers and parents in order to win competitions, simultaneously repressing their needs for playing with friends or for developing their own potentialities (Cho-Han, 2000). As a result, people are accepting workaholism as a shortcut to get recognized as a productive person. Finally, the socio-economic discrepancies among people or groups repeatedly bring about the feeling of “comparative deprivation” along with the feeling of emptiness in life. These feelings can easily make them bound to workaholism (Schor, 1992), as they feel, in the midst of workaholism, relieved of anxiety or depression. Besides, they can even dream that workaholism will help them overcome poverty or comparative deprivation, illusionary as it is. In brief, it is remarkable that under workaholism there lies deep-rooted fear resulting from traumatic experiences, individually or collectively: the fear of death, of failure, and of emptiness (cf. Schaef, 1987; Fassel, 1990; Burke, 2000; Heide, 2001).

Further, the binary logistic regression provided information about causality between socio-demographic variables and workaholism. Those in their 40s, male workers, and working longer hours, and involuntary job takers are more inclined to workaholism as compared to each reference group in both crude and adjusted analyses. This result is in line with some previous studies (Snir and Harpaz, 2009; Ariapooran, 2019) and different from others (Kemeny, 2002; Andreassen et al., 2014), except for the relationship of (in)voluntariness in choosing employment type with workaholism, which is discussed later.

However, job security did not show any significant effect on workaholism in the adjusted analysis, though it was significant in the crude one. In contrast, education level indicated a significant positive association with workaholism in the adjusted analysis, while showing no significance in the crude one. Finally, the marital status and income level were consistently insignificant in predicting workaholism in both analyses. This implies that both the poor and the rich have their own internal drive to work excessively: for example, the poor can pacify their pain of life by working addictively, whereas the rich tend to stimulate their feeling of self-realization through workaholism.

Besides, one could measure workaholism not as a binary variable but as a continuous one, especially considering the *progressive* character of workaholism (Schaef, 1987). In this study, the K-WAQ ranged between 1 and 5 and its mean and standard deviation was 2.31 and 0.51, respectively. Based on this continuous measurement, either t-test or ANOVA presented how different the workaholism level of each group was. Above all, six socio-demographic variables effectively showed the difference in workaholism among sub-groups, except for the two variables, namely, marital status and income level. This result is in line with some precedent research (Burke, 1999; Buelens and Poelmans, 2004; Snir and Harpaz, 2009), and might differ from the evidence presented for other cultures (Burke et al., 2004; Burgess et al., 2006). In terms of gender, men indicated higher workaholism than women in Korea, too (Kanai et al., 1996; Snir and Harpaz, 2009), which can be different in Western countries (Burke, 1999; Andreassen et al., 2014). Moreover, married men showed the highest workaholic tendency followed by unmarried women, married women, and unmarried men. Those with job security showed higher workaholism than those without security. Further, those *involuntary* job takers exhibited higher workaholism than the voluntary ones. In terms of work hours, those working more than 50 h per week had higher workaholism than those working 40 h or less per week. The 30s and 40s age groups displayed higher workaholism than the 50s and 60s ones. Lastly, those with university diplomas exhibited higher workaholism than those with high school or middle school attainment.

Overall, four variables, namely, gender, age, work hours and involuntariness in employment revealed themselves as significant predictors of workaholism in Korea, very consistently through all the models in this study (Tables 6, 11). Male workers tend to be more exposed to workaholism than female. Those in their 40s are more addicted to work than the elderly. The work hours were positively and the voluntariness in choosing employment type was negatively associated with workaholism, each with statistical significance.

While the rationale why work hours are positively related to workaholism is apparent in precedent research (Schaufeli et al., 2008), that of (in)voluntariness in choosing employment type, to date, is unclear. One possible reason would be that those who had to take their job – either with job security or without it – involuntarily, are likely to try to show salient performance to their supervisors in order to freely choose another job with better characteristics such as skill variety or autonomy (cf. Hackman and Oldham, 1975). This explanation can be in line with the findings of Sharma et al. (2017), which highlighted that in UAE, for instance, the lack of job autonomy tends to “transpire to workaholism.” Moreover, it is notable that non-permanent workers in South Korea are exposed to highly poor working conditions in comparison to permanent workers, although the Korean labor laws prohibit discrimination and inequality in terms of wage, job stability, and fringe benefits (Kim and Lee, 2014). Therefore, those who involuntarily took their job as non-permanent are likely to show more workaholic tendency, trying to make a good impression to be recognized by supervisors as self-sacrificing and loyal contributors to their organization. In contrast, those who involuntarily took their job as permanent workers, for example, because of financial difficulties, can easily become workaholics out of fear of becoming losers in the performance-oriented society.

## Conclusion

The current study had two objectives: to develop a Korean form of workaholism measure (K-WAQ) by elaborating the WAQ developed originally by Aziz et al. (2013), and to investigate, based upon the K-WAQ, divergent workaholic tendencies in Korea by using a nationally representative data for the first time. Through this two-phased research the workaholism prevalence in South Korea (39.7%) as well as its differences according to socio-demographic variables was demonstrated.

The theoretical and methodological contributions of this study can be summarized as follows. First, it provides a validation of WAQ in the Korean context, by developing K-WAQ (Tables 1, 3). Although many researchers have meanwhile tried to develop a Korean version of workaholism scale (cf. Oum and Lee, 2018; Seo et al., 2018; Yoon, 2018), they mainly tried to apply the WAQ to Korean society (and that just in Korean language). In fact, they did not provide an adequate measure to estimate workaholism in Korea. Consequently, they failed to examine the reliability as well as the validity of new construct in a systematic way. The current study is the first paper in English both to develop the K-WAQ in a systematic way and to estimate the Korean workaholism prevalence and its differences among socio-economic groups. In so doing, this study makes a methodological contribution to the applicability of WAQ (Aziz et al., 2013) to populations across different cultures and contexts other than the United States. This study provides evidence that the five-factor structure of the 29-item WAQ as suggested by Aziz et al. (2013) is not sustainable, at least for Korea (Table 5). However, the Korean form of 15-item K-WAQ (four-factor structure) shows adequate validity as well as significant reliability (Tables 3, 4).

Second, in this study, workaholism prevalence and differences among sub-groups in terms of socio-demographic variables are predicted through statistical analyses such as *t*-test, ANOVA, GLM, and binary logistic regressions (Tables 6–9). Above all, the workaholism prevalence in South Korea was statistically estimated to 39.7% (Table 9).

Further, this prevalence of workaholism in Korea was differentiated according to socio-demographic characteristics. Specifically, the mean difference analyses detected that men, permanent workers, involuntary job-takers, long-hour workers, workers with university diplomas, and those in the age group of the 30s and 40s have more workaholic tendencies in Korea. Besides, binary logistic analyses along with GLM provided some significant correlates of workaholism: (married) men, the number of work hours per week, the involuntariness in choosing employment type, and the 40s age group provided clear indications of workaholic tendencies among South Korean employees (Tables 7, 8, and 11). Notably, the interaction effect between gender and marital status was also confirmed through this study (Tables 7, 8).

Third, the fact that married men (followed subsequently by unmarried women, married women, and unmarried men) have the highest level of workaholic tendencies has a special implication. Despite increasing women’s participation in economic activities in Korea (57% in 2014; Song, 2016), the role of bread-winner is often played by married men. It implies that patriarchal culture is still highly prevalent in Korea, although the Gender Equality Law in Korea has been in effect since 1988. That the age group of 40s has higher odds than others implies that much attention should be paid to this cluster in order to protect them from the *karoshi*, death from overwork (cf. North and Morioka, 2016). Interestingly, there was neither marital status nor income effect on the level of workaholism, when analyzed independently. This indicates that people in poverty, as well as affluent people, are evenly exposed to workaholism in Korea, which implies a need for a comprehensive and universal welfare programs (Woo, 2011).

In summary, the present study, as an initial step exploring workaholism in Korea, clarifies that South Korean workers carry considerable risk of workaholism. Its prevalence of 39.7% legitimizes calling South Korea *a workaholic society*.

This aspect also presents another practical implication: workaholic tendencies among Korean workers would, in turn, negatively affect labor productivity, not to speak of health. According to OECD statistics from 2014^5^, the Korean labor productivity (GDP divided by average annual hours worked) amounted to 30.4 US dollars (working 2,076 hours), while that of Norway, for example, was 87.1 dollars (working 1,427 hours). In addition to individual healing from workaholism, a systemic efforts for work time reduction and, at the same time, for change of working processes (Heide, 2009), seem to be highly urgent in the workaholic society of Korea.

Further, a relatively high workaholism prevalence in Korea, with its differentiation depending on socio-demographic characteristics, implies the existence of a variety of antecedents, mediators, and moderators promoting workaholism in Korea. Beside urgent need for proper treatment and intervention strategies, further theoretical, as well as empirical work, is necessary. Therefore, prospective researchers could deep dive into the subject to intensively examine the underlying mechanisms leading to and sustaining workaholism in Korea. The present study provides preliminary cues toward this end. Especially societal as well as organizational contexts seem to be highly relevant for future investigation.

## Limitations and Future Research Directions

This study has also certain limitations. First, it focused primarily on several socio-demographic variables. Future research should elucidate, beyond demographic differences, familial, organizational, and societal predictors of workaholism. Secondly, this study had extreme difficulty in interpreting “neutral” in the 5-point Likert scale. The 5-point Likert scale would be more appropriate in evaluating things value-laden. For measuring workaholism, rather a 4-point scale might be better to obtain clarity in assessing workaholism. Third, in this study, workaholism was measured by K-WAQ in a continuum from 1 to 5 via face-to-face interview. Therefore, the results may be exposed to common method bias (Fuller et al., 2016). Further, measuring workaholism through self-reporting can be highly restrictive in analyzing reality, as the respondents can abhor being stigmatized as workaholics. Thus, future research might need a longitudinal study design with qualitative research methods to closely observe the underlying insidious *processes* of workaholism. Fourth, this study offers only some academic information about Korean workaholism. To expand and deepen our understanding of workaholism and to find out cultural-specific intervention strategies, periodic international comparative research based on a consistent and unified definition of workaholism is needed. In addition, other measures than the WAQ such as DUWAS or BWAS are to validate in the Korean context, as they seem more universal than the WAQ. Such efforts will help us surmount any possible cultural bias in the measure developed in a specific socio-cultural context.

Despite these limitations, the present study is expected to contribute to the international academic community on workaholism by developing K-WAQ and providing preliminary analyses on workaholism prevalence and its differences in South Korea.

## Data Availability Statement

Publicly available datasets were analyzed in this study. This data can be found here: https://www.kli.re.kr/klips_eng/index.do.

## Ethics Statement

Ethical review and approval was not required for the study on human participants in accordance with the local legislation and institutional requirements. The patients/participants provided their written informed consent to participate in this study.

## Author Contributions

SK is the only author to analyze data and create the manuscript in a final form as it is. Thus he is also the corresponding author. SK contributed to the article and approved the submitted version.

## Conflict of Interest

The author declares that the research was conducted in the absence of any commercial or financial relationships that could be construed as a potential conflict of interest.

## Footnotes

1 https://stats.oecd.org/

2 http://kostat.go.kr

3 https://www.kli.re.kr/klips_eng/index.do

4 http://kostatis.go.kr

5 https://stats.oecd.org/

## References

[B1] Aldahadha B. (2019). The level of workaholism and its relation to the positive and negative perfectionism. *Pol. Psychol. Bull.* 50 157–166.

[B2] Andreassen C. S. (2014). Workaholism: An overview and current status of the research. *J. Behav. Addict.* 3 1–11. 10.1556/JBA.2.2013.017 25215209 PMC4117275

[B3] Andreassen C. S. Griffiths M. D. Hetland J. Pallesen S. (2012). Development of a work addiction scale. *Scand. J. Psychol.* 53 265–272. 10.1111/j.1467-9450.2012.00947.x 22490005

[B4] Andreassen C. S. Griffiths M. D. Hetland J. Kravina L. Jensen F. Pallesen S. (2014). The prevalence of workaholism: a survey study in a nationally representative sample of Norwegian employees. *PLoS One* 9:e102446. 10.1371/journal.pone.0102446 25118877 PMC4131865

[B5] Andreassen C. S. Schaufeli W. B. Pallesen S. (2018). Myths about “the myths about work addiction” commentary on: ten myths about work addiction (Griffiths et al., 2018). *J. Behav. Addict.* 7 858–862. 10.1556/2006.7.2018.126 30556780 PMC6376365

[B6] Andreassen C. S. Ursin H. Eriksen H. R. (2007). The relationship between strong motivation to work, “workaholism”, and health. *Psychol. Health* 22 615–629. 10.1080/14768320600941814

[B7] Ariapooran S. (2019). Sleep problems and depression in Iranian nurses: the predictive role of workaholism. *Iran. J. Nurs. Midwifery Res.* 24 30–37. 10.4103/ijnmr.ijnmr_188_17 30622575 PMC6298169

[B8] American Psychiatric Association (APA) (2013). *Diagnostic and Statistical Manual of Mental Disorders.* Washington, DC: American Psychiatric Publishing.

[B100] Atroszko P. A. Demetrovics Z. Griffiths M. D. (2019). Beyond the myths about work addiction: toward a consensus on definition and trajectories for future studies on problematic overworking: a response to the commentaries on: ten myths about work addiction (Griffiths et al., 2018). *J. Behav. Addict.* 8, 7–15. 10.1556/2006.8.2019.11 30920291 PMC7044606

[B9] Aziz S. Uhrich B. Wuensch K. L. Swords B. (2013). The workaholism analysis questionnaire: emphasizing work-life imbalance and addiction in the measurement of workaholism. *J. Behav. Appl. Manag.* 14 71–86.

[B10] Aziz S. Zamary S. Wuensch K. (2018). The endless pursuit for self–validation through attainment: an examination of self–esteem in relation to workaholism. *Pers. Individ. Dif.* 121 74–79. 10.1016/j.paid.2017.09.024

[B11] Aziz S. Zickar M. J. (2006). A cluster analysis investigation of workaholism as a syndrome. *J. Occup. Health Psychol.* 11 52–62. 10.1037/1076-8998.11.1.52 16551174

[B12] Balkin R. S. Reiner S. M. Hendricks L. Washington A. McNeary S. Juhnke G. A. (2018). Life balance and work addiction among African Americans. *Career Dev. Q.* 66 77–84. 10.1002/cdq.12123

[B13] Brown R. I. F. (1993). “Some contributions of the study of gambling to the study of other addictions,” in *Gambling Behavior and Problem Gambling*, eds Eadington W. R. Cornelius J. J. (Reno, NV: University of Nevada Press), 241–272.

[B14] Buelens M. Poelmans S. A. (2004). Enriching the Spence and Robbins’ typology of workaholism: demographic, motivational and organizational correlates. *J. Organ. Chang. Manag.* 17 440–458. 10.1108/09534810410554470

[B15] Burgess Z. Burke R. J. Oberklaid F. (2006). Workaholism among Australian psychologists: gender differences. *Equal. Divers. Incl. Int. J.* 25 48–59. 10.1108/02610150610645968

[B16] Burke R. J. (1999). Workaholism in organizations: gender differences. *Sex Roles* 41 333–345.

[B17] Burke R. J. (2000). Workaholism in organizations: the role of personal beliefs and fears. *Anxiety Stress Coping* 13 53–64. 10.1080/10615800008248333

[B18] Burke R. J. Oberklaid F. Burgess Z. (2004). Workaholism among Australian women psychologists: antecedents and consequences. *Women Manag. Rev.* 19 252–259. 10.1108/09649420410545971

[B19] Campbell D. T. Fiske D. W. (1959). Convergent and discriminant validation by the multitrait–multimethod matrix. *Psychol. Bull.* 56 81–105. 10.1037/h0046016 13634291

[B20] Carroll J. J. Robinson B. E. (2000). Depression and parentification among adults as related to parental workaholism and alcoholism. *Fam. J.* 8 360–367. 10.1177/1066480700084005

[B21] Cho-Han H. (2000). ‘You are entrapped in an imaginary well’: the formation of subjectivity within compressed development-a feminist critique of modernity and Korean culture. *Inter Asia Cult. Stud.* 1 49–69. 10.1080/146493700360999

[B22] Clark M. A. Lelchook A. M. Taylor M. L. (2010). Beyond the big five: how narcissism, perfectionism, and dispositional affect relate to workaholism. *Pers. Individ. Dif.* 48 786–791. 10.1016/j.paid.2010.01.013

[B23] D’Sa J. L. Visbal–Dionaldo M. L. (2017). Analysis of multiple choice questions: item difficulty, discrimination index and distractor efficiency. *Int. J. Nurs. Educ.* 9 109–114. 10.5958/0974-9357.2017.00079.4

[B24] Fabrigar L. R. Wegener D. T. MacCallum R. C. Strahan E. J. (1999). Evaluating the use of exploratory factor analysis in psychological research. *Psychol. Methods* 4 272–299. 10.1037/1082-989x.4.3.272

[B25] Fassel D. (1990). *Working Ourselves to Death: The High Cost of Workaholism, the Rewards of Recovery.* San Francisco, CA: Harper.

[B26] Feldman J. (2016). The simplicity principle in perception and cognition. *Wiley Interdiscip. Rev. Cogn. Sci.* 7 330–340. 10.1002/wcs.1406 27470193 PMC5125387

[B27] Flowers C. P. Robinson B. (2002). A structural and discriminant analysis of the work addiction risk test. *Educ. Psychol. Meas.* 62 517–526. 10.1177/001316402128774941

[B28] Fuller C. M. Simmering M. J. Atinc G. Atinc Y. Babin B. J. (2016). Common methods variance detection in business research. *J. Bus. Res.* 69 3192–3198. 10.1016/j.jbusres.2015.12.008

[B29] Gorgievski D. M. Bakker A. (2010). “Passion for work: work engagement versus workaholism,” in *Handbook of Employee Engagement*: Perspectives, issues, research and practice, ed. Albrecht S. L. (London: Edward Elgar Publishing), 264–271.

[B30] Griffiths M. D. (2005). A ‘components’ model of addiction within a biopsychosocial framework. *J. Subst. Use* 10 191–197. 10.1080/14659890500114359

[B31] Griffiths M. D. (2011). Workaholism: a 21st century addiction. *Psychol. Bull. Br. Psychol. Soc.* 24 740–744.

[B32] Griffiths M. D. Demetrovics Z. Atroszko P. A. (2018). Ten myths about work addiction. *J. Behav. Addict.* 7 845–857. 10.1556/2006.7.2018.05 29409339 PMC6376361

[B33] Hackman J. R. Oldham G. R. (1975). Development of the job diagnostic survey. *J. Appl. Psychol.* 60 159–170. 10.1037/h0076546

[B34] Hamilton Skurak H. Malinen S. Näswall K. Kuntz J. C. (2018). Employee well–being: the role of psychological detachment on the relationship between engagement and work–life conflict. *Econ. Ind. Democracy* 26 10.1177/0143831X17750473

[B35] Han K.-H. (2011). Workaholism and socio-cultural context. *Korean J. Manag. Stud. (Korean)* 24 2519–2547.

[B36] Heide H. (2001). Individual and social economic dimensions of work addiction. *Paper Delivered at the Congress “Sucht 2000” Which was Held by the German Central Bureau Against Dangers of Addiction in Karlsruhe, 13th-15th November 2000*, Germany.

[B37] Heide H. (2009). Globalization of the work society. proposal for a re-interpretation of the work society as a posttraumatic syndrome. *Trans Humanit.* 1 9–38. 10.1353/trh.2009.0001

[B38] Kanai A. Wakabayashi M. Fling S. (1996). Workaholism among employees in Japanese corporations: an examination based on the Japanese version of the workaholism scales. *Jpn Psychol. Res.* 38 192–203. 10.1111/j.1468-5884.1996.tb00024.x

[B39] Kang S.-D. (2000). Labour relations in Korea between crisis management and living solidarity. *Inter-Asia Cult. Stud.* 1 393–407. 10.1080/14649370020009906

[B40] Kang S.-D. (2003). An exploratory study on the theory and evidence of work addiction. *Korean J. Labor Stud. (Korean)* 9 197–234.

[B41] Kang S.-D. (2020). *A Critical Analysis of the WAQ: Development of a Korean Form.* Available online at: https://advance.sagepub.com/articles/A_critical_analysis_of_the_WAQ_Development_of_a_Korean_Form/11876541 (accessed May 18, 2020). 10.1177/0033294120959779 32985356

[B42] Kemeny A. (2002). Driven to excel: a portrait of Canada’s workaholics. *Can. Soc. Trends* 64 2–7.

[B43] Killinger B. (1991). *Workaholics: The Respectable Addicts.* Toronto, ON: Key Porter Books.

[B44] Kim A. E. Lee C. M. (2014). Neoliberalism and insecure employment in Korea: emergence of the working poor and worsening socio-economic polarization. *Korea Obs.* 45 255–273.

[B45] Kunecka D. Hundert M. (2019). The extent of workaholism in a group of polish nurses. *Int. J. Health Plan. Manage.* 34 e194–e202. 10.1002/hpm.2636 30156712

[B46] Lanzo L. Aziz S. Wuensch K. (2016). Workaholism and incivility: stress and psychological capital’s role. *Int. J. Workplace Health Manag.* 9 165–183. 10.1108/ijwhm-08-2015-0051

[B47] Lee G.-Y. Geum J.-H. An J.-Y. (2015). *Report on Basic Analyses of the 17th Wave Korean Labor and Income Panel Study (in Korean).* Seoul: Korea Labor Institute.

[B48] Lemmens J. S. Valkenburg P. M. Peter J. (2009). Development and validation of a game addiction scale for adolescents. *Media Psychol.* 12 77–95. 10.1080/15213260802669458

[B49] Li C. H. (2016). Confirmatory factor analysis with ordinal data: comparing robust maximum likelihood and diagonally weighted least squares. *Behav. Res. Methods* 48 936–949. 10.3758/s13428-015-0619-7 26174714

[B50] MacCallum R. Widaman K. Zhang S. Hong S. (1999). Sample size in factor analysis. *Psychol. Methods* 4 84–99. 10.1037/1082-989x.4.1.84 26822184

[B51] Machlowitz M. (1980). *Workaholics, Living With Them, Working With Them.* Reading, MA: Addison Wesley Publishing Company.

[B52] Mazzetti G. Biolcati R. Guglielmi D. Vallesi C. Schaufeli W. B. (2016). Individual characteristics influencing physicians’ perceptions of job demands and control: the role of affectivity, work engagement and workaholism. *Int. J. Environ. Res. Public Health* 13:567. 10.3390/ijerph13060567 27275828 PMC4924024

[B53] McMillan L. H. W. O’Driscoll M. P. Marsh N. V. Brady E. C. (2001). Understanding workaholism: data synthesis, theoretical critique, and future design strategies. *Int. J. Stress Manag.* 8 69–91.

[B54] McMillan L. H. W. Brady E. C. O’Driscoll M. P. Marsh N. V. (2002). A multifaceted validation study of Spence and Robbins’ (1992) workaholism battery. *J. Occup. Organ. Psychol.* 75 357–368. 10.1348/096317902320369758

[B55] Moyer F. Aziz S. Wuensch K. (2017). From workaholism to burnout: psychological capital as a mediator. *Int. J. Workplace Health Manage.* 10 213–227. 10.1108/ijwhm-10-2016-0074

[B56] Mudrack P. E. (2006). “Understanding workaholism: the case of behavioral tendencies,” in *Research Companion to Working Time and Work Addiction*, ed. Burke R. J. (Cheltenham: Edward Elgar Publishing), 108–128.

[B57] Ng T. W. Sorensen K. L. Feldman D. C. (2007). Dimensions, antecedents, and consequences of workaholism: a conceptual integration and extension. *J. Organ. Behav.* 28 111–136. 10.1002/job.424

[B58] Nonnis M. Cuccu S. Cortese C. G. Massidda D. (2017). The Italian version of the Dutch Workaholism Scale (DUWAS): a study on a group of nurses. *Bollettino di Psicologia Applicata [BPA–Appl. Psychol. Bull.]* 65 47–57.

[B59] North S. Morioka R. (2016). Hope found in lives lost: karoshi and the pursuit of worker rights in Japan. *Contemp. Jpn.* 28 59–80. 10.1515/cj-2016-0004

[B60] Oates W. E. (1971). *Confessions of a Workaholic: The Facts About Work Addiction.* New York, NY: World Publishing Company.

[B61] Oum S. Lee J. (2018). Hanguk geunrojaeui iljungdok [workaholism among Korean employees]. Hanguk Gajeong Gwanri Hakhoiji. *J. Korean Home Manag. Assoc.* 36 1–23. 10.1007/978-3-030-15697-8_1

[B62] Porter G. (1996). Organizational impact of workaholism: suggestions for researching the negative outcomes of excessive work. *J. Occup. Health Psychol.* 1 70–84. 10.1037/1076-8998.1.1.70 9547036

[B63] Porter G. (2001). Workaholic tendencies and the high potential for stress among co-workers. *Int. J. Stress Manag.* 8 147–164.

[B64] Porter L. Steers R. Mowday R. Boulian P. (1974). Organizational commitment, job satisfaction, and turnover among psychiatric technicians. *J. Appl. Psychol.* 59 603–609. 10.1037/h0037335

[B65] Pratarelli M. E. Browne B. L. (2002). Confirmatory factor analysis of internet use and addiction. *CyberPsychol. Behav.* 5 53–64. 10.1089/109493102753685881 11990975

[B66] Quinones C. Griffiths M. D. (2015). Addiction to work: a critical review of the workaholism construct and recommendations for assessment. *J. Psychosoc. Nurs. Ment. Health Serv.* 53 48–59. 10.3928/02793695-20150923-04 26489104

[B67] Raubenheimer J. (2004). An item selection procedure to maximize scale reliability and validity. *SA J. Ind. Psychol.* 30 59–64.

[B68] Roberson R. B. III Elliott T. R. Chang J. E. Hill J. N. (2014). Exploratory factor analysis in rehabilitation psychology: a content analysis. *Rehabil. Psychol.* 59 429–438. 10.1037/a0037899 25221958

[B69] Robinson B. E. (1998/2013). *Chained to the Desk: A Guidebook for Workaholics, Their Partners and Children, and the Clinicians Who Treat Them*, 3rd Edn New York, NY: NYU Press.

[B70] Ryan R. M. Deci E. L. (2000). Self-determination theory and the facilitation of intrinsic motivation, social development, and well-being. *Am. Psychol.* 55 68–78. 10.1037/0003-066x.55.1.68 11392867

[B71] Schaef A. W. (1987). *When Society Becomes an Addict.* New York, NY: Harper Collins.

[B72] Schaef A. W. Fassel D. (1988). *The Addictive Organization.* New York, NY: Harper & Row Publishers.

[B73] Schaufeli W. B. Shimazu A. Taris T. W. (2009). Being driven to work excessively hard. The evaluation of a two-factor measure of workaholism in the Netherlands and Japan. *Cross Cult. Res.* 43 320–348. 10.1177/1069397109337239

[B74] Schaufeli W. B. Taris T. W. Van Rhenen W. (2008). Workaholism, burnout, and work engagement: three of a kind or tree different kinds of employee well-being? *Appl. Psychol. Int. Rev.* 57 173–203. 10.1111/j.1464-0597.2007.00285.x

[B75] Schor J. (1992). *The Overworked American: The Unexpected Decline of Leisure.* New York, NY: Basic Books.

[B76] Scott K. S. Moore K. S. Micelli M. P. (1997). An exploration of the meaning and consequences of workaholism. *Hum. Relations* 50 287–314. 10.1177/001872679705000304

[B77] Seo E. Jeong Y. Park S. (2018). Iljungdok profilebyul yeonghyang yoin. [an empirical study on workaholism using latent profile analysis]. Haenjeong Nonchong. *Korean J. Policy Stud.* 56 221–253. 10.24145/kjpa.56.4.8

[B78] Sharma J. Singh A. Sharma P. (2017). A journey into the heart of workaholism from cross-cultural perspective. *Int. J. Hum. Resour. Dev. Manag.* 17 185–204. 10.1504/ijhrdm.2017.10007431

[B79] Snir R. Harpaz I. (2009). Workaholism from a cross-cultural perspective. *Cross Cult. Res.* 43 303–308. 10.1177/1069397109336987

[B80] Song J. (2016). Activating women in the labor market: the development of south korea’s female-friendly employment and labor market policies. *Korea Obs.* 47 559–596.

[B81] Spence J. T. Robbins A. S. (1992). Workaholism: definition, measurement, and preliminary results. *J. Pers. Assess.* 58 160–178. 10.1207/s15327752jpa5801_15 16370875

[B82] Stoeber J. Davis C. R. Townley J. (2013). Perfectionism and workaholism in employees: the role of work motivation. *Pers. Individ. Dif.* 55 733–738. 10.1016/j.paid.2013.06.001

[B83] Sussman S. Lisha N. Griffiths M. (2011). Prevalence of the addictions: a problem of the majority or the minority? *Eval. Health prof.* 34 3–56. 10.1177/0163278710380124 20876085 PMC3134413

[B84] Urbán R. Kun B. Mózes T. Soltész P. Paksi B. Farkas J. (2019). A four-factor model of work addiction: the development of the work addiction risk test revised. *Eur. Addict. Res.* 25 145–160. 10.1159/000499672 30982051

[B85] Van Beek I. Hu Q. Schaufeli W. B. Taris T. W. Schreurs B. H. (2012). For fun, love, or money: what drives workaholic, engaged, and burned−out employees at work? *Appl. Psychol.* 61 30–55. 10.1111/j.1464-0597.2011.00454.x

[B86] Villella C. Martinotti G. Di Nicola M. Cassano M. La Torre G. Gliubizzi M. D. (2011). Behavioural addictions in adolescents and young adults: results from a prevalence study. *J. Gambl. Stud.* 27 203–214. 10.1007/s10899-010-9206-0 20559694

[B87] World Health Organization (WHO) (1992). *The ICD-10 Classification of Mental and Behavioral Disorders. Clinical and descriptions and diagnostic guidelines.* Geneva: World Health Organization.

[B88] Woo M. (2011). A newly emerging small welfare state and social cleavage: the Korean case. *Korea Obs.* 42 645–676.

[B89] Yoon J. (2018). Iljungdok cheukjeonggwa siltae. [measurement and state of workaholism among Korean workers]. Saneup Nodong Yeongoo. *Korean J. Labor Stud.* 24 229–260.

[B90] Zaiţ A. Bertea P. S. P. E. (2011). Methods for testing discriminant validity. *Manag. Mark. J.* 9 217–224.

